# Why you should Mini-Med School: Mini-Med School as an intervention to increase health literacy

**DOI:** 10.36834/cmej.67817

**Published:** 2020-12-07

**Authors:** Sergiy Shatenko, Samuel Harder, Jane Gair

**Affiliations:** 1 Island Medical Program, University of Victoria, British Columbia, Canada; 2 Faculty of Medicine, University of British Columbia, British Columbia, Canada

## Abstract

**Background:**

Health literacy is an increasingly important topic in healthcare given that low health literacy is widely prevalent and linked to poorer health outcomes and higher healthcare costs. We sought to determine if a Mini-Med School delivered by medical students could prove to be an effective intervention to improve health literacy in the elderly.

**Methods:**

This study took place in the context of the University of British Columbia Medical Doctorate Undergraduate Program’s Flexible and Enhanced Learning course. It aimed to evaluate the effectiveness of a Mini-Med School lecture series as an intervention to increase health literacy in 24 volunteer participants from the University of Victoria Retirees Association. This was a cross sectional study comparing health literacy pre- and post-intervention using the validated Health Literacy Questionnaire.

**Results:**

There was a statistically significant improvement in seven of nine scales of health literacy when participants repeated the Health Literacy Questionnaire six weeks post-intervention as well as positive outcomes from both a student learning and community outreach perspective.

**Discussion:**

This study demonstrates that a Mini-Med School program is an effective way to increase health literacy; adds to the minimal research surrounding Mini-Med Schools; and should further encourage Canadian medical schools to use Mini-Medical Schools as a method of engagement and advocacy with their communities.

## Abstract (fr)

**Contexte:**

La littératie en santé est un sujet gagnant en importance dans les soins de santé, puisqu’un faible niveau de littératie est très prévalent et relié à des issues de santé défavorables et à des coûts de soins de santé plus élevés. Nous avons cherché à savoir si une mini-école de médecine offerte par des étudiants en médecine pourrait être une intervention efficace pour améliorer la littératie en santé chez les personnes âgées

**Méthodologie:**

Cette étude s’est déroulée dans le cadre d’un cours d’apprentissage par projet faisant partie du programme de premier cycle en médecine de l'Université de Colombie-Britannique. Elle visait à évaluer l’efficacité d’une série de conférences de la Mini-école de médecine comme intervention pour augmenter les la littératieen santé chez 24 participants volontaires de l’association des retraités de l’Université de Victoria. Il s'agissait d'une étude transversale visant à comparer la littératie en santé avant et après l’intervention à l’aide du questionnaire validé Health Literacy Questionnaire.

**Résultats:**

On a observé une amélioration statistiquement significative dans sept des neuf échelles de littératie en santé lorsque les participants ont répondu de nouveau au questionnaire six semaines après l’intervention, ainsi que des résultats positifs tant du point de vue de l’apprentissage des étudiants que de la sensibilisation de la collectivité.

**Discussion:**

Cette étude démontre qu’un programme de mini-écoles de médecine est un moyen efficace d’accroître la littératie en santé; elle alimente la littérature actuellement limitée portant sur les mini-écoles de médecine; elle devrait inciter davantage d’écoles de médecine canadiennes à utiliser les mini-écoles de médecine comme méthode de mobilisation et de défense des intérêts dans leurs collectivités.

## Introduction

An estimated 60% of Canadians (88% of seniors) have poor health literacy. An abundance of research over the past three decades has emerged to demonstrate the prevalence of low health literacy and its consequences including poorer health outcomes, increased use of health care services and lower self-reported health.^1^^-^^5^^,^^7^^,^^8^ Given the high prevalence and the associated negative outcomes, interventions are needed to improve health literacy.^9^^-^^17^ Possible interventions such as workshops, radio shows and peer support groups have been highlighted in the 2014 Canadian report “Examples of Health Literacy in Practice”^37^ based on their ability to address key domains of health literacy including access, comprehension, evaluation and communication.

An additional domain of health literacy, collaborative learning and social support, was identified by De Wit. et al. (2017) as a key practice that contributes to health literacy in the elderly and their communities. One possible strategy that has the potential to address these key domains and to make use of collaborative learning and that has yet to be fully investigated is the use of Mini-Med Schools (MMS); a series of lectures on medical topics presented to the public by students or faculty from a medical school or hospital. Medical faculties and some community hospitals across Canada have used MMS programs as community outreach tools with the proposed outcomes of empowering the public to take ownership of their health, breaking down barriers between the public and medical professionals, and providing information on basic medical science. These proposed outcomes make MMS a prime candidate as an intervention to improve health literacy and a method for medical schools to actively engage in improving the health literacy of their communities.

In this study we aimed to demonstrate that an MMS is an effective intervention to improve health literacy and thus add to the research surrounding MMS.

## Methods

This was a cross-sectional study using a sample of volunteer participants.

### Recruitment

We recruited the participants for the MMS Lecture Series using a sample from the University of Victoria (UVic) Retiree Association’s Elder Academy (UVRAEA) consisting of retired UVic faculty and staff, along with their significant others. Recruitment via email included the initial letter of contact and a consent form outlining the nature of the study. Participation in the study was voluntary and was not a prerequisite to attending the lecture series. Study participants were required to attend a minimum of four out of six lectures.

### Program format

The two-hour lectures ran weekly from January 14 to February 18, 2017 at UVic. In consultation with the UVRAEA program chair, two second year medical students created and presented evidence-based lectures on Cardiovascular Health, Preventative Medicine, Brain Health, Drugs and Polypharmacy, Medical Testing, and Navigating the Health Care System. The lectures were interactive and included pre-lecture tests and opportunities to answer questions and discuss anecdotes. The interactive portion included questions throughout the presentations, as well as dedicated question and discussion periods.

### Assessment of intervention

To measure the impact of our intervention, we chose the Health Literacy Questionnaire (HLQ), a validated health literacy measurement tool designed by Deakin University. ^32^

The HLQ measures nine scales of health literacy with themes related to obtaining, understanding, appraising and using health information, as well as healthcare provider support and engagement, social supports, and ability to navigate the healthcare system. Scales 1-5 are measured on a 4-point Likert-type scale and scales 6-9 are measured on a 5-point Likert-type scale. The specific details of each scale cannot be released as per our agreement with Deakin University but the titles of each scale can be found in Table 2. Participants filled out a copy of the HLQ prior to the first lecture and six to eight weeks after the last lecture.

**Table 1:** Participant characteristics

Participant characteristics	
Age, mean (SD), years	72.9 (7.98)
Sex; No. (%)	
female	16 (67)
male	8 (33)
Lives alone; No. (%)	
yes	9 (38)
no	15 (62)
Country of birth; No. (%)	
Canada	14 (58)
other	10 (42)
Education Completed; No. (%)	
High School	4 (16)
Trade	1 (4)
Undergraduate	9 (38)
Postgraduate	10 (42)
Longstanding Illness or disability; No. (%)	
Arthritis	9 (38)
Back pain	5 (21)
Heart disease	7 (29)
Asthma	4 (17)
Depression/Anxiety	5 (17)
Cancer	3 (13)
Diabetes	0 (0)
Stroke	0 (0)
Other	8 (33)
Have visited the Emergency Department in the last year; No. (%)	9 (38)

**Table 2:** Pre and post intervention results

	Pre-Intervention scores		Post-Intervention Scores		Wilcoxon Signed-Rank Test		Effect Size
**Scale**	Mean (SD)	Median	Mean (SD)	Median	Z	p-value (2-tailed)	Cohen’s *d*
**1**	3.10 (0.70)	3	3.40 (0.44)	3.5	-3.30	0.001^*^	0.51
**2**	2.50 (0.45)	2.5	2.74 (0.40)	2.75	-2.38	0.017^*^	0.55
**3**	3.08 (0.39)	3	3.22 (0.39)	3.2	-2.07	0.038^*^	0.36
**4**	2.83 (0.60)	3	3.01 (0.50)	3	-1.79	0.073	0.33
**5**	2.72 (0.41)	2.8	2.95 (0.38)	3	-2.84	0.005^*^	0.58
**6**	3.62 (0.87)	4	3.91 (0.43)	4	-2.17	0.03^*^	0.44
**7**	3.33 (0.72)	3.5	3.67 (0.50)	3.83	-2.86	0.004^*^	0.55
**8**	3.58 (0.42)	3.6	3.70 (0.38)	3.8	-1.95	0.051	0.30
**9**	3.86 (0.53)	3.8	4.04 (0.49)	3.8	-2.52	0.012^*^	0.35

Scale 1: Feeling understood and supported by healthcare providers. Scale 2: Having sufficient information to manage my health care. Scale 3: Actively managing my health. Scale 4: Social support for health. Scale 5: Appraisal of health information. Scale 6: Ability to actively engage with healthcare providers. Scale 7: Navigating the healthcare system. Scale 8: Ability to find good health information. Scale 9: Understand health information well enough to know what to do. Statistically significant results are denoted with an asterisk (p < 0.05).

### Data analysis

The pre-intervention HLQ results of each participant were compared to the post-intervention HLQ results using a paired Wilcoxon Signed-Rank Test. Each of the domains measured by the HLQ was analyzed independently. Those participants that did not complete both pre- and post-intervention questionnaires were excluded from the analysis. A two-tailed p-value of 0.05 was used. Cohen’s *d* test was used to calculate the effect size for all nine of the scales.

### Ethics

The ethics approval for this project was granted by the University of British Columbia (UBC) Behavioral Research Ethics Board (BREB) under the UBC Class based ethics application for the UBC Medical Doctorate Undergraduate Program (MDUP) Flexible and Enhanced Learning (FLEX) course.

### Setting

This project was part of the FLEX course in the UBC MDUP curriculum. FLEX is a course designed to allow medical students to pursue scholarly activities during their undergraduate medical education. It was made possible through collaboration with Let’s Talk Science (LTS), a non-profit organisation dedicated to science outreach, and the UVRAEA, a program that promotes physical and mental health for seniors through educational experiences.

## Results

A total of 60 individuals signed up for the lecture series of which 40 enrolled in the study and completed the pre-intervention HLQ. Sixteen participants did not complete the post-intervention survey and were excluded from the study, leaving 24 data sets available for analysis. The study participants were highly educated, with 80% having at least an undergraduate degree. Most also had one or more long-standing illnesses (Table 1).

There were statistically significant improvements in the scores for seven of the nine scales measured by the HLQ with effect sizes ranging from 0.35-0.58 in these seven scales (Table 2). The only two areas where the improvement was not significant was in social support for health and ability to find good health information.

## Discussion

The consequences of the high prevalence of low health literacy in Canada range from negative impacts on individual health to increased system wide costs and patient volumes. Interventions exist to address this problem, but further interventions are still needed. This study aimed to demonstrate that an MMS may be an effective intervention to improve health literacy and add to the minimal research surrounding the outcomes of MMS programs.

Using the Health Literacy Questionnaire, a statistically significant increase was seen in each of seven of the nine health literacy scales measured by the HLQ. The effect size for these improvements was small to moderate (0.35-0.58) for these seven scales. This result is practically important with positive implications for medical schools and community hospitals in that an MMS is a low-cost, simple intervention that serves not only as a means of community engagement but a method by which medical practitioners, learners and educators can easily engage in improving health literacy. The magnitude of the effect size in the scales with a statistically significant improvement is impressive considering the simplicity and ease of implementation of the intervention.

A potential explanation for the effectiveness of the MMS is its ability to address key domains of health literacy. An MMS mirrors some of the successful interventions seen in the report “Examples of Health Literacy in Practice” by addressing access, comprehension and understanding.

Scales 2, 8 and 9 of the HLQ capture access and comprehension of health information, and scales 1 and 6 capture communication of health from the patient perspective. Access to and comprehension of health information was increased through presentation of various relevant medical topics using plain language and audio and visual aids. This increase in access and comprehension is reflected in score increases in scale 2 and 9 respectively. The informal and regular contact with presenting health care professionals and encouragement to ask questions likely increased participants’ ability to and comfort with communicating with health professionals leading to an increase in communication scales 1 and 6. Scale 8 did not see an improvement in scores as finding reliable health information was not addressed directly but could be integrated in future MMS initiatives.

Another important aspect of MMS intervention is its social nature. De Wit et al. (2017) found, in a meta-analysis of community-based initiatives to improve health literacy, that social support and collaborative learning were key practices that contribute to health literacy in the elderly and their communities.^33^ Given this result, the social interaction aspects of the MMS, including meeting weekly with a large group of participants and discussions between participants, may contribute to the success of an MMS as well.

There were limitations to this study. The sample was a small, self-selected group of highly educated participants, and we are unable to comment on the long-term effects of the intervention. As well, given that upon enrollment the participants were aware of the intervention and its intent, this stimulated behaviours that would lead to increased health literacy and affect results of the post-intervention survey. Other behaviours that have a positive impact on health literacy, such as improved confidence when interacting with health care professionals, and being more interested in health information, may have been stimulated through the MMS and may explain the scores. These are indirect benefits of the MMS that can be at least in part attributed to the intervention. These were not explored in this study but could be included in future studies.

Further research could be done to formally investigate the effect of an MMS on feelings of community engagement and social involvement. This would be an important piece of evidence to add, since both social connectedness and social capacity have been shown to be associated with higher levels of health literacy.^13^ In addition, future MMS lecture series could be expanded to serve other at-risk populations.^18^ Other potentially important benefits to consider, which were not formally part of the purpose of this study, but were noted by the medical students involved in the project, included improved public speaking skills and communication of complex medical topics in a clear and concise manner.^34^ This is significant as physician communication skills have been promoted as a key strategy to improve health literacy in patients and is a core CanMEDS competency.^23^^,^^35^

## Conclusion

Although this study is small in size and does have limitations, it adds to the research illustrating that an MMS could serve as a potential simple, low cost intervention for addressing low health literacy; a growing issue with numerous known negative outcomes. In addition, and important for medical educators, it shows leadership in fulfilling the contract between doctors and society to not only provide medical care but to educate and engage with the community as a whole.
